# Supplementary material for: Comparison of Recruitment Patterns of Sessile Marine Invertebrates According to Substrate Characteristics
Source: Int J Environ Res Public Health. 2022 Jan 19;19(3):1083. doi: 10.3390/ijerph19031083 (PMC8834478; doi:10.3390/ijerph19031083)
Supplement: Supplementary file 1 [file ijerph-19-01083-s001.zip › ijerph-1512793-supplementary.pdf]

Table S1. Species lists for the ascidian group and the bryozoan group.

Group	Ascidian		Bryozoan	
	**Solitary**	**Colonial**	**Branching**	**Encrusting**
Species list	*Ascidiella aspersa*<br>*Ciona robusta*<br>*Ciona savignyi*<br>*Molgula manhattensis*<br>*Halocynthia aurantium*<br>*Halocynthia roretzi*<br>*Styela clava*<br>*Styela plicata*	*Didemnum vexillum.*<br>*Symplegma* sp.<br>*Botryllus schlosseri*<br>*Botrylloides violaceus*	*Bugula neritina*<br>*Bugulina californica*<br>*Tricellaria occidentalis*	*Celleporaria brunnea*<br>*Jellyella tuberculata*<br>*Schizoporella unicornis*<br>*Watersipora subtorquata*

Table S2. Cumulative precipitation (mm) and precipitation ratio (%) observed during the survey period in the three sites.

Date		MP		TY		BS	
2016	Sep	197.8	43.4%	543.3	47.6%	407.9	43.3%
	Dec	45.9	10.0%	118.1	10.3%	103.8	11.0%
2017	Mar	28.5	6.2%	32.6	2.9%	35.7	3.8%
	Jun	28.5	6.2%	68.4	6.0%	49.8	5.3%
	Sep	109.4	24.0%	362.2	31.7%	335.0	35.5%
	Dec	45.3	9.9%	17.6	1.5%	10.6	1.1%
Total		455.4	100.0%	1142.2	100.0%	942.8	100.0%

Table S3. Tide levels (cm) at the three sites observed during the study period.

Site	Minimum	Maximum	Average	SD
MP	189.9	459.5	254.9	20.1
TY	118.8	184.2	152.4	12.7
BS	47.5	105.0	74.17	10.5

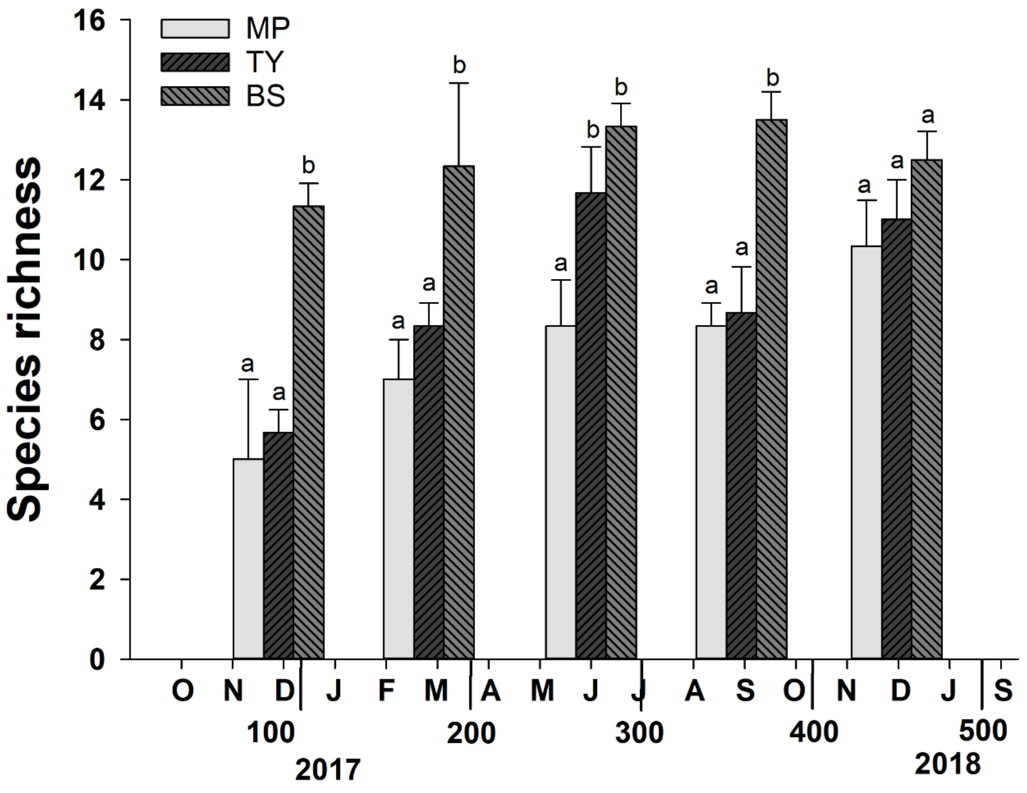

Figure S1. Species richness observed at three sites during the study period. Standard deviation bars with different letters are statistically different (p < 0.05); ab, no statistically significant difference between letters a and b (p > 0.05).

Table S4. Results of MRPP analysis of the three survey sites using mean coverage. Bold values denote significant results.

Groups compared	Test statistic (T)	Chance-corrected within-group agreement (A)	*p*–value
MP × TY	−3.38311	0.20909	**0.00369**
MP × BS	−0.26704	0.01454	0.36589
TY × BS	−2.57200	0.13090	**0.00979**

Table S5. PERMANOVA and PERMDISP pair-wise comparison results for differences in substrates and sites using the mean coverage of taxa.

Variable		PERMANOVA		PERMDISP	
		t	*P*(perm)	t	*P*(perm)
Site	MP vs. TY	2.522	**0.000**	1.798	0.104
	MP vs. BS	1.567	0.050	2.414	**0.020**
	TY vs. BS	2.144	**0.004**	0.899	0.404
Substrate	Rubber vs. Stone	1.368	0.127	0.627	0.592
	Rubber vs. Tarpaulin	0.918	0.474	0.094	0.940
	Rubber vs. Iron	0.731	0.687	1.786	0.133
	Stone vs. Tarpaulin	1.298	0.146	0.642	0.586
	Stone vs. Iron	1.530	0.079	2.382	**0.046**
	Tarpaulin vs. Iron	0.615	0.784	1.541	0.214

Table S6. SIMPER analysis of taxa between each substrate in the three survey sites

Site	Substrate	Taxa	Average abundance		Average dissimilarity	Dissimilarity SD	Contribution %
			A	B			
Mokpo	Stone vs. Rubber	Colonial ascidian	1.54	3.74	14.39	1.26	25.67
		Bivalvia	2.03	2.47	12.85	0.98	22.93
		Cirripedia	2.64	2.07	8.12	1.09	14.48
	Stone vs. Tarpaulin	Colonial ascidian	3.74	1.38	16.1	1.26	26.02
		Bivalvia	2.47	2.02	13.45	0.93	21.74
		Cirripedia	2.07	1.97	9.23	1.22	14.91
	Stone vs. Iron	Colonial ascidian	3.74	1.33	15.73	1.29	25.65
		Bivalvia	2.47	2.24	13.48	0.97	21.98
		Cirripedia	2.07	2.89	10.53	1.52	17.16
	Rubber vs. Tarpaulin	Cirripedia	2.64	1.97	14.63	0.85	23.31
		Bivalvia	2.03	2.02	13.55	0.96	21.6
		Colonial ascidian	1.54	1.38	9.75	0.98	15.55
	Rubber vs. Iron	Cirripedia	2.64	2.89	15.02	0.9	24.85
		Bivalvia	2.03	2.24	13.55	1.00	22.42
		Colonial ascidian	1.54	1.33	9.53	0.96	15.77
	Tarpaulin vs. Iron	Cirripedia	1.97	2.89	17.62	1.01	27.42
		Bivalvia	2.02	2.24	14.28	0.94	22.23
		Colonial ascidian	1.38	1.33	8.73	1.08	13.58

Table S6 Continued

Site	Substrate	Taxa	Average abundance		Average dissimilarity	Dissimilarity SD	Contribution %
			A	B			
Tong yeong	Stone vs. Rubber	Branched Bryozoa	2.86	3.04	8.64	1.26	20.81
		Bivalvia	1.60	2.47	7.91	1.23	19.03
		Cirripedia	4.92	5.08	6.72	0.75	16.17
	Stone vs. Tarpaulin	Bivalvia	2.47	1.77	7.63	1.27	19.95
		Branched Bryozoa	3.04	2.78	6.71	1.23	17.53
		Solitary ascidian	1.75	2.02	5.33	1.22	13.94
	Stone vs. Iron	Bivalvia	2.47	1.53	7.76	1.31	18.51
		Cirripedia	5.08	3.95	7.5	0.97	17.88
		Branched Bryozoa	3.04	2.71	7.16	1.29	17.07
	Rubber vs. Tarpaulin	Branched Bryozoa	2.86	2.78	9.08	1.28	21.64
		Cirripedia	4.92	4.56	6.76	0.81	16.10
		Bivalvia	1.60	1.77	6.58	1.16	15.67
	Rubber vs. Iron	Branched Bryozoa	2.86	2.71	9.71	1.13	21.8
		Cirripedia	4.92	3.95	8.81	0.89	19.78
		Solitary ascidian	1.56	2.00	6.65	1.07	14.94
	Tarpaulin vs. Iron	Branched Bryozoa	2.78	2.71	7.60	1.37	18.46
		Cirripedia	4.56	3.95	7.10	1.01	17.25
		Bivalvia	1.77	1.53	6.33	1.24	15.38

Table S6
Continued

Site	Substrate	Taxa	Average abundance		Average dissimilarity	Dissimilarity SD	Contribution %
			A	B			
Busan	Stone vs. Rubber	Bivalvia	2.57	1.8	10.47	0.88	19.75
		Cirripedia	1.97	2.86	10.39	1.04	19.59
		Colonial ascidian	2.42	1.77	9.88	1.14	18.63
	Stone vs. Tarpaulin	Bivalvia	1.77	3.47	11.72	1.21	24.75
		Colonial ascidian	2.86	2.27	9.04	1.01	19.08
		Cirripedia	1.80	2.27	8.19	1.23	17.29
	Stone vs. Iron	Cirripedia	1.8	3.12	12.33	1.28	22.35
		Colonial ascidian	2.86	2.16	11.47	0.91	20.78
		Bivalvia	1.77	2.21	9.27	1.18	16.8
	Rubber vs. Tarpaulin	Bivalvia	2.42	3.47	11.71	1.2	25.33
		Cirripedia	2.57	2.27	8.97	1.22	19.4
		Colonial ascidian	1.97	2.27	7.52	1.03	16.25
	Rubber vs. Iron	Cirripedia	2.57	3.12	13.31	1.12	24.44
		Bivalvia	2.42	2.21	10.65	1.13	19.56
		Colonial ascidian	1.97	2.16	9.12	0.96	16.75
	Tarpaulin vs. Iron	Bivalvia	3.47	2.21	12.15	1.17	24.43
		Cirripedia	2.27	3.12	11.15	1.4	22.41
		Colonial ascidian	2.27	2.16	7.83	1	15.75

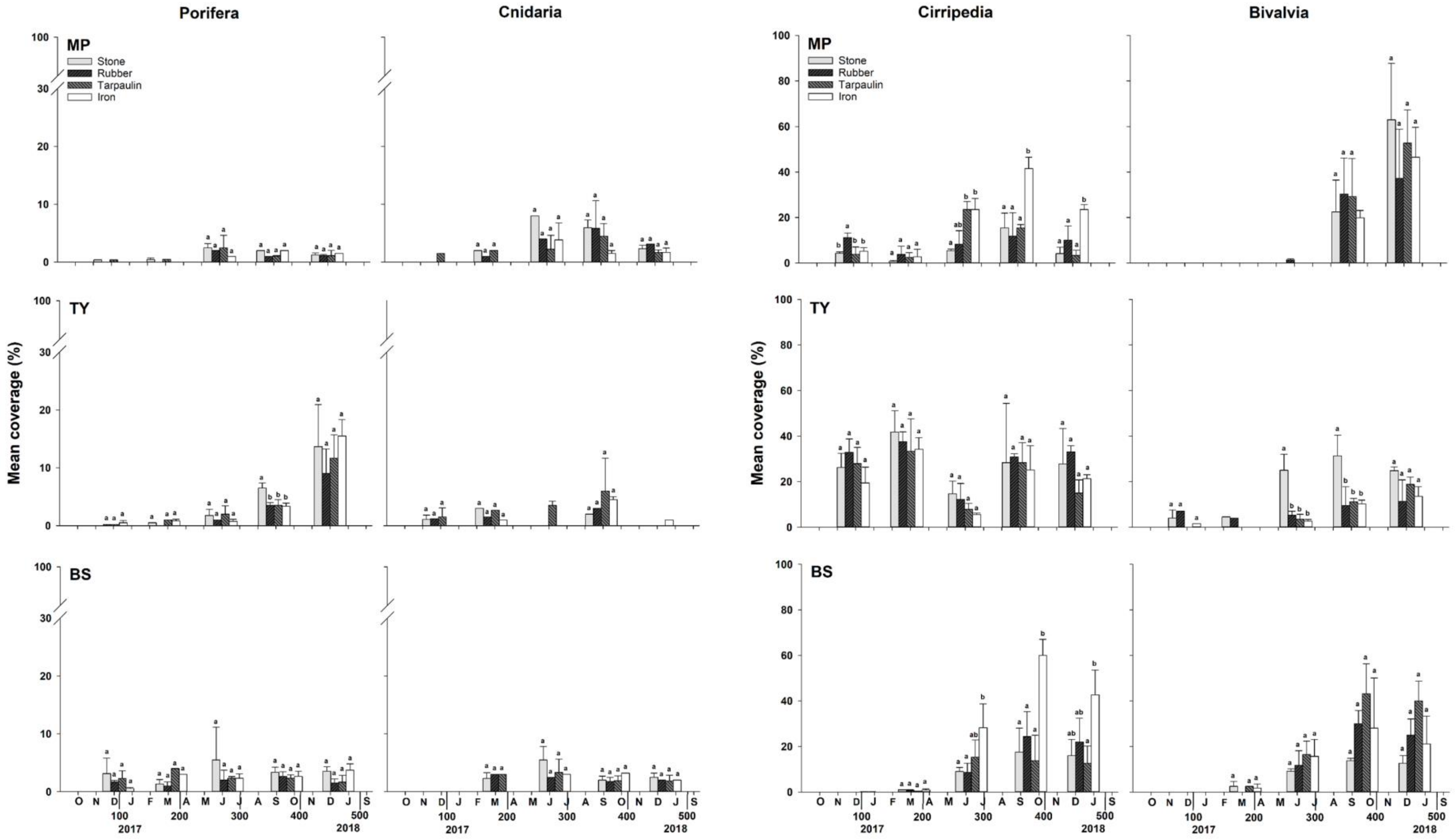

Figure S2. Mean coverage of eight taxa in each survey site. Standard deviation bars with different letters are statistically different ($p < 0.05$); ab, no statistically significant difference between letters a and b ($p > 0.05$).

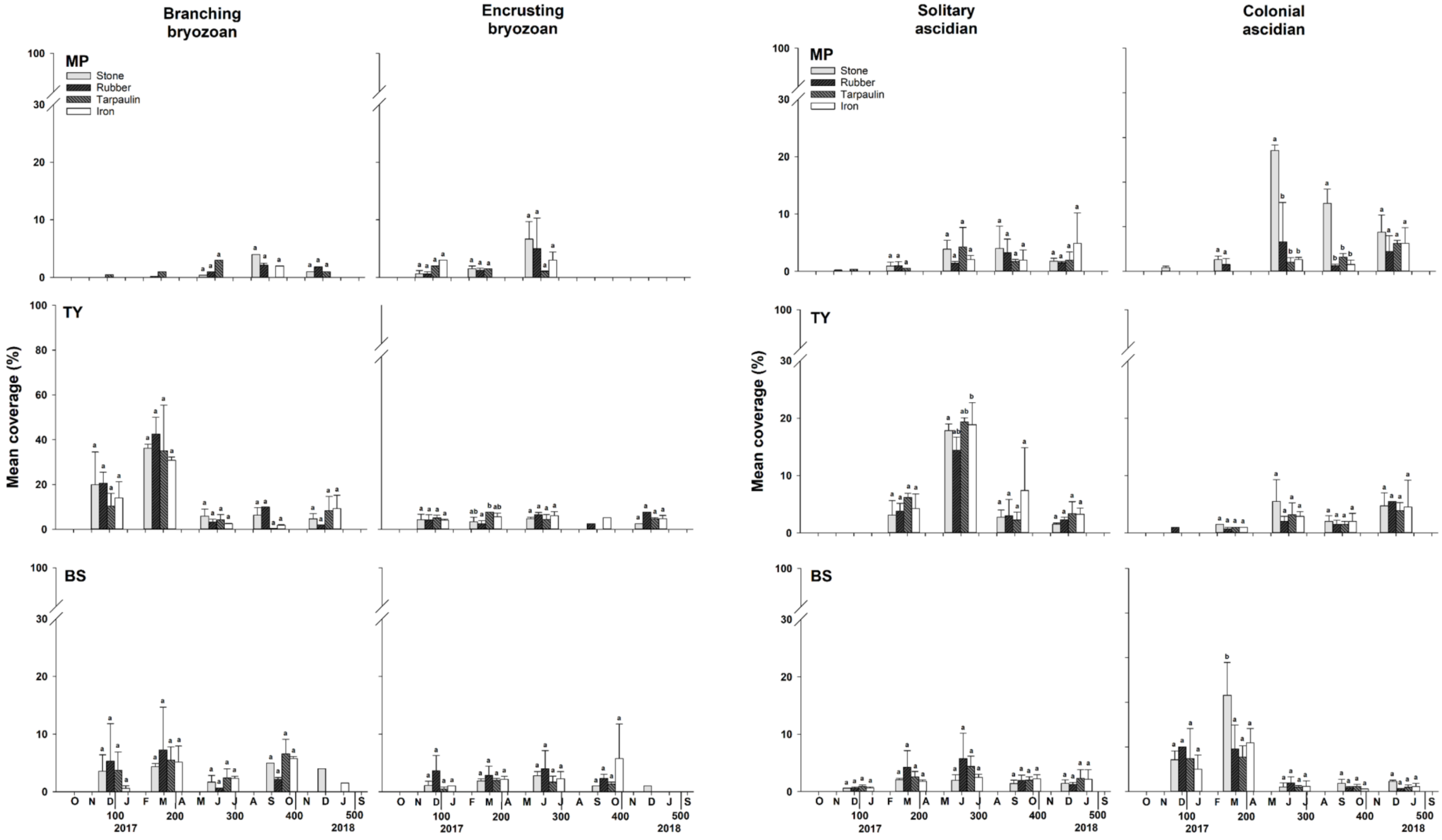

Figure S2. Continued.

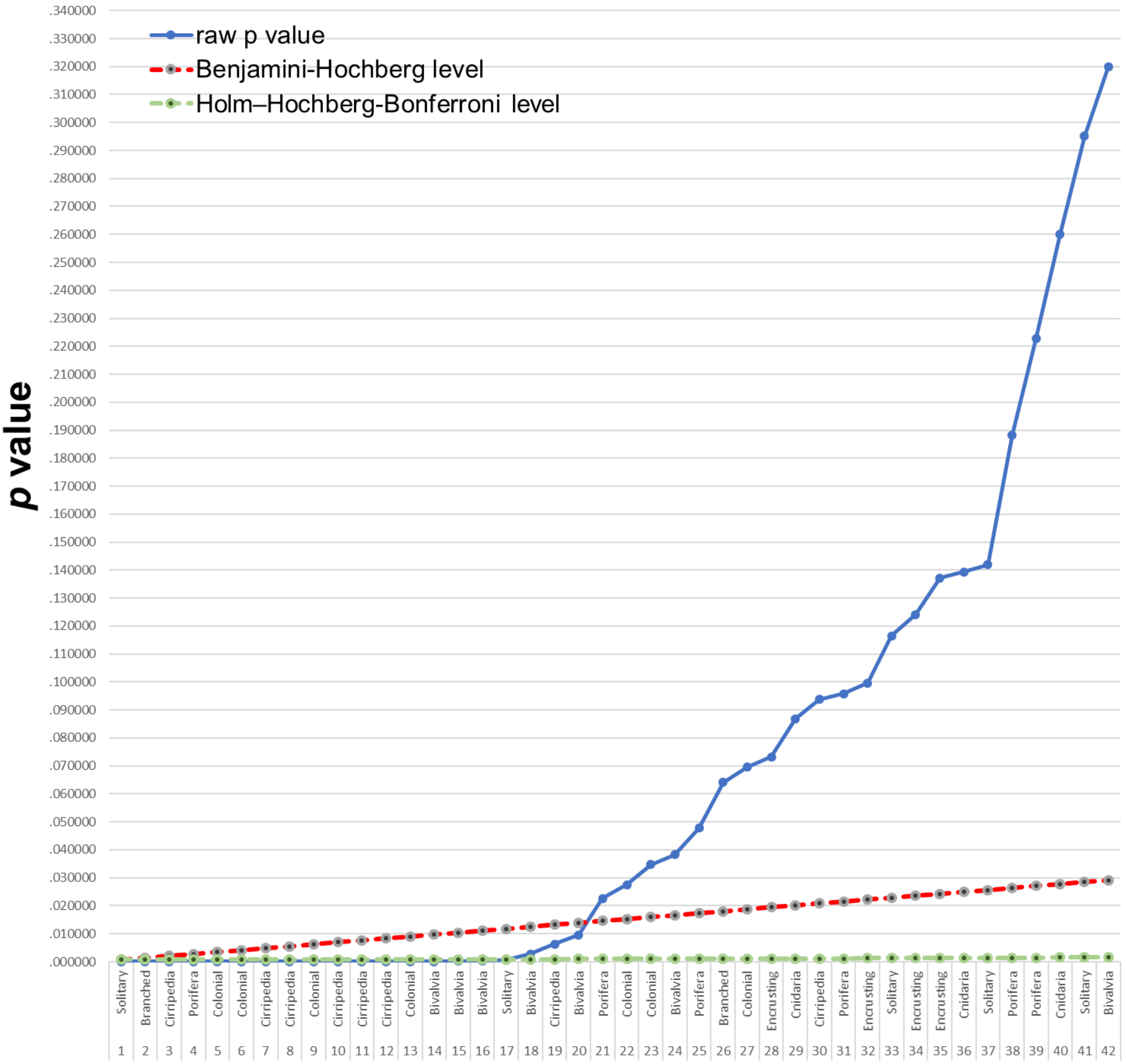

Figure S3. Result of Benjamini and Hochberg (1995) FDR control. The graph results show 72 ranked raw *p*-values, with only 20 raw p-values corresponding to the 0.05 FDR level.

Table S7. List of sessile marine invertebrates observed from survey sites during the study period. Asterisks indicate rare species (i.e., those occurring with a frequency of < 5 %) excluded from analysis.

Taxa \ Site	Mokpo	Tongyeong	Busan
Phylum Porifera			
Class Demospongiae			
Order Haplosclerida			
Family Chalinidae			
Haliclona (*Reniera*) *cinerea* *	○	○	○
Order Suberitida			
Family Halichondriidae			
Halichondria (*Halichondria*) *bowerbanki*	○	○	○
Phylum Cnidaria			
Class Anthozoa			
Order Actiniaria			
Family Actiniidae			
Anthopleura kurogane	○	○	○
Class Hydrozoa			
Order Anthoathecata			
Family Tubulariidae			
Ectopleura crocea			○
Order Leptothecata			
Family Campanulariidae			
*Obelia dichotoma**	○	○	
Phylum Mollusca			
Class Bivalvia			
Order Mytilida			
Family Mytilidae			
Mytilus galloprovincialis	○	○	○
Order Ostreida			
Family Ostreidae			
Magallana gigas	○	○	○
Phylum Arthropoda			
Class Hexanauplia			
Order Sessilia			
Family Balanidae			
Amphibalanus amphitrite	○		
*Amphibalanus eburneus**		○	○
Amphibalanus improvisus			○
Balanus trigonus	○	○	○
*Megabalanus rosa**			○

Table S5

Continued

Taxa \ Site	Mokpo	Tongyeong	Busan
Phylum Bryozoa			
Class Gymnolaemata			
Order Cheilostomatida			
Family Bugulidae			
Bugula neritina	○	○	○
Bugulina californica	○	○	○
Family Candidae			
Tricellaria occidentalis	○	○	○
Family Lepraliellidae			
Celleporaria brunnea	○	○	○
Family Membraniporidae			
*Jellyella tuberculata**		○	○
Family Schizoporellidae			
Schizoporella unicornis			○
Family Watersiporidae			
Watersipora subtorquata	○	○	○
Phylum Chordata			
Class Ascidiacea			
Order Aplousobranchia			
Family Didemnidae			
Didemnum vexillum.	○	○	○
Order Phlebobranchia			
Family Ascidiidae			
Ascidiella aspersa	○	○	○
Family Cionidae			
Ciona robusta	○	○	○
Ciona savignyi	○		
Order Stolidobranchia			
Family Molgulidae			
Molgula manhattensis	○	○	
Family Pyuridae			
*Halocynthia aurantium**			○
Halocynthia roretzi	○	○	○
Herdmania momus			○

Family Styelidae			
Styela clava	○	○	○
Styela plicata	○	○	○
Symplegma sp.		○	○
Botryllus schlosseri	○	○	○
Botrylloides violaceus	○	○	○
